# Oxidative Stress and Cognitive Decline: The Neuroprotective Role of Natural Antioxidants

**DOI:** 10.3389/fnins.2021.729757

**Published:** 2021-10-13

**Authors:** Ferdinando Franzoni, Giorgia Scarfò, Sara Guidotti, Jonathan Fusi, Muzaffar Asomov, Carlo Pruneti

**Affiliations:** ^1^Department of Clinical and Experimental Medicine, University of Pisa, Pisa, Italy; ^2^Department of Medicine and Surgery, University of Parma, Parma, Italy

**Keywords:** oxidative stress, cognitive decline, natural antioxidants, neurodegenerative diseases, neuroprotection

## Abstract

Free- radicals (Oxygen and Nitrogen species) are formed in mitochondria during the oxidative phosphorylation. Their high reactivity, due to not-engaged electrons, leads to an increase of the oxidative stress. This condition affects above all the brain, that usually needs a large oxygen amount and in which there is the major possibility to accumulate “Reacting Species.” Antioxidant molecules are fundamental in limiting free-radical damage, in particular in the central nervous system: the oxidative stress, in fact, seems to worsen the course of neurodegenerative diseases. The aim of this review is to sum up natural antioxidant molecules with the greatest neuroprotective properties against free radical genesis, understanding their relationship with the Central Nervous System.

## Introduction

Oxidative stress is known to be involved in the pathogenesis of several diseases: in particular, a strict connection between a free-radical increase and the onset of neurodegenerative disorders has been widely demonstrated (Migliore and Coppedè, 2009).

Free radicals are atoms or molecules characterized by one or more electrons not engaged in chemical bonds, which, remaining unpaired, tend to accept electrons from other molecules: this reaction causes their oxidation (Harman, 1956; Valko et al., 2007). An oxidation–reduction imbalance in living organisms leads to an excess of reactive oxygen and nitrogen species (RONS) with a consequent oxidative stress status (Valko et al., 2007; Sies, 2015) that is classified as basal, low, intermediate, and high according to its intensity (Kishida and Klann, 2007; Lushchak, 2014).

There is a large number of antioxidant defensive mechanisms against RONS. The antioxidant molecules are divided into two groups: enzymatic and non-enzymatic compounds. The enzymatic group includes superoxide dismutase (SOD), catalase (CAT), glutathione peroxidase (GPx) and glutathione reductase (GR). SOD, one of the main protective mechanisms against ROS, catalyzes the conversion of O2- to H_2_O_2_ and O_2_ (Halliwell and Gutteridge, 1984), while CAT converts the generated H_2_O_2_ into water and O_2_ (Rodriguez-Rocha et al., 2013). The non-enzymatic group involves glutathione (GSH), abundant in brain cells, thioredoxin (Trx), vitamins A, E and C, selenium, retinoic acid, carotenoids, and flavonoids. GSH reacts with ROS to generate glutathione disulfide (GSSG) and enters a cycle together with GPx and GR (Cenini et al., 2019).

All these systems are essential to protect us against a possible free radical damage. Since the brain consumes a large amount of oxygen (about 20% more than other parts of the body), if antioxidant defenses are insufficient and levels of polyunsaturated lipids are high, there will be the possibility of an accumulation of biomolecules damaged by RONS (Wang et al., 2012). So, neuronal cells are particularly vulnerable to oxidative damage because of their high oxygen consumption, the weak antioxidant defense (Cobley et al., 2018) and high content of polyunsaturated fatty acids in their membranes: in fact, the lipids of the neuronal membrane are rich in chains side polyunsaturated fatty acids (PUFA). PUFAs composed of eicosapentaenoic (C20:5) and decosahexanoic (C22:6) acids are particularly vulnerable to free radicals attack due to the double bonds that allow RONS to remove hydrogen ions (Hawkins et al., 1998).

In particular, RONS overproduction in brain cells reacts with cell membrane PUFAs causing their peroxidation (Rahman, 2007). More specifically, lipid peroxidation generates a heterogeneous group of relatively stable products such as malondialdehyde (MDA), 4-hydroxy-2-nonenal (HNE), acrolein and isoprostane (Reed, 2011).

As a result, membrane fluidity decreases causing a greater permeability. This facilitates a massive entry of substances into the intracellular system (e.g., K+, Ca2+, etc.), that could alter membrane proteins, enzymes and receptors (Fukuzawa and Gebicki, 1983).

Carbohydrates are also influenced by RONS with the formation of advanced glycation products (AGE) (Gabbita et al., 1998) involved in the development of neurodegenerative disorders (Ahmed, 2005).

In addition, RONS alter DNA and RNA heterocyclic bases, in particular guanine: these alterations occur in Parkinson’s disease (PD) affected brains. Instead, Alzheimer’s Disease (AD) affected brains, are characterized by elevated carbonylation and nitration, that respectively, introduce in proteins carbon monoxide or one or more NO_2_ groups derived from nitric acid (Alam et al., 1997; Ahmed, 2005).

All neurodegenerative disorders share several common characteristics, such as an abnormally aggregated protein accumulation and mitochondrial dysfunction that demonstrate an oxidative stress status (Abramov et al., 2017). In particular, neurodegeneration-involved reactive species are hydrogen peroxide (H_2_O_2_), superoxide anion (O_2_^–^) and highly reactive hydroxyl radical (HO ⋅) (Cooke et al., 2003). They are able to preclude the protein reduction, cause translation errors *in vivo* altering protein structure, and function (Dukan et al., 2000).

In addition, Nitric Oxide (NO) appears to play an important role in neurological disorders. It has one unpaired electron that makes it highly susceptible to other molecules. Released into the bloodstream, it is oxidized to form nitrite and nitrate (Lundberg et al., 2008; Tewari et al., 2021). The synthesis of NO is regulated by Nitric Oxide Synthase (NOS) that, in the human body exists in three forms: inducible nitric oxide synthase (iNOS), neuronal nitric oxide synthase (nNOS) and the endothelial nitric oxide synthase (eNOS). The amount of NO, produced by these different isoforms, shows a different physiological activity. At low concentrations, NO seems to have a neuroprotective effect: studies in animals model showed that NOS inhibition correlated with the genesis and the progression of PD, and with a decreased neuronal apoptosis (Steinert et al., 2010). Nevertheless, NO at high concentrations, induces a proinflammatory stimulus with a neurotoxic effect (Good et al., 1998; Tse, 2017): a study conducted on PD affected brains, demonstrated that NO and peroxynitrite were involved in the degeneration of neurons in the substantia nigra pars compacta (Moncada and Higgs, 1993).

The risk of developing neurodegenerative disorders is also related to some lifestyle factors, such as obesity, sedentary lifestyle, and unbalanced diet, because of their role in RONS genesis (Tan et al., 2018; Nuzzo et al., 2019).

Therefore, considering the fact that oxidative stress is one of the most important risk factors involved in the onset, maintenance and progression of neurodegenerative diseases, a healthy and balanced diet, with its consequent intake of natural antioxidants, could have a fundamental protective role against them (Steele, 2007; Johri and Beal, 2012; Kumar and Ratana, 2016; Khan et al., 2018).

**FIGURE 1:**
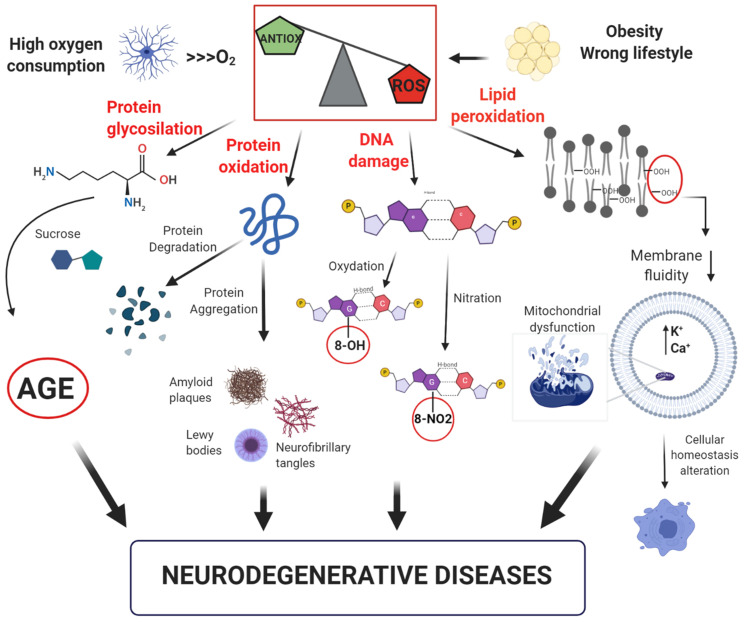
Model of free-radical formation and its consequences at a cellular level. The intense oxygen consumption in the brain induces the formation of reactive oxygen species (ROS). Their high reactivity leads to an increase of the oxidative stress, which promotes: (i) glycosylation and oxidation of proteins, leading to the formation of advanced glycation products (AGE) or loss of protein function; (ii) DNA damage with oxidation or nitration of guanine bases; (iii) lipid peroxidation with reduction of membrane fluidity and increase in cell permeability, resulting in alteration of cellular homeostasis. All these factors can contribute to the development of neurodegenerative disorders.

The oxidative stress theory and its consequences at cellular level is shown in Figure 1.

## Vitamin C and E

A diet characterized by vegetables and fruits, usually rich in Vitamin C, carotenoids, and Vitamin E, is positively associated with cognitive efficiency and reduced the risk of dementia in the elderly.

From a chemical point of view, Vitamin C is defined as Ascorbic Acid (AA). It has six-carbon compound that contain two acid-ionizing groups (Ballaz and Rebec, 2019). In the human body, the brain is the region with the highest concentration of AA (Smythies, 1996). This high concentration, attests to the fundamental involvement of AA in the brain function. Indeed, many studies suggest that AA has a neuroprotective role thanks to an antioxidant activity modulation (Harrison et al., 2010a,b). This modulation is related to the buffering of the oxidizing species induced by methamphetamine (Ito et al., 2007), homocysteine (Machado et al., 2011), ethanol (Tian et al., 2016) and other molecules (Gudelsky, 1996; Stansley and Yamamoto, 2014).

It is interesting to note that the AA activity is quite vast, also considering the interaction with Vitamin E. Their association is remarkable in the protection of membranes and other hydrophobic compartments (Beyer, 1994; Getoff, 2013).

A clinical study has highlighted the association between vitamin E and C intake and a delayed AD onset in a group of elderly subjects (Shen and Ji, 2012); similar results were also obtained by Shen and colleagues in 2012 (Kontush and Shekatolina, 2004). In fact, it has been shown that a supplementation of these vitamins and so their greater concentration in cerebrospinal fluids can prevent lipid oxidation in AD patients (Taghizadeh et al., 2017).

The importance of vitamin C in preventing and combating neurological disorders has also been demonstrated in a recent work: in a murine model, decreased levels of AA levels influenced the neural network development, and this alteration correlated with the pathophysiology of neurological disorders (Ikeda et al., 2021).

In an *in vitro* study, Lee et al. (2021) investigated the protective effect of AA administration in preventing age-induced oxidative damage in hippocampal neurons, demonstrating that a regular AA treatment protected hippocampal neurons from free radical damages.

Vitamin E is a lipophilic molecule that could be found in plants and in many Mediterranean diet food (Schirinzi et al., 2019). Vit. E is referred to compounds called tocopherols and tocotrienols (Ulatowski and Manor, 2015). These usually include eight molecules (α-, β-, γ-, δ-tocopherols and α-, β-, γ-,δ -tocotrienols), with great antioxidant capacity (Jiang, 2014).

The presence of an electrophilic hydroxyl group on the chroman ring, allows Vitamin E to be a strong antioxidant. To understand Vitamin E role as a protective factor in neurodegenerative disorders, it must be considered what happens if it is deficient. For example, it is demonstrated that Vitamin E deficit is related to an impairment of cerebellar Purkinje neurons that are the main integrators of cerebellar neural circuits (Ulatowski and Manor, 2015). As far as PD, evidence suggests that a Vitamin E supplementation can improve symptoms, functional capabilities and the inflammatory state of affected patients (Simonetto et al., 2019).

In addition, Khanna et al. (2003) showed a fundamental role of Vitamin E against glutamate- induced neurotoxicity. In a later study, it is observed that the co-treatment with vitamin E analogs can block NO or O2⋅ donor-induced cell death in rat striatal cultures (Osakada et al., 2004).

Therefore, the use of vitamins E and C as antioxidant supplements is fundamental to delay the onset of neurodegenerative disorders and their complications.

## Fatty Acids

Recently, it has grown an interest in polyunsaturated fatty acids (PUFAs) and their beneficial effects on health, due to their strong antioxidant properties (Fotuhi et al., 2009; Sokoła-Wysoczańska et al., 2018). PUFAs (omega-3 and omega- 6 fatty acids) usually have two or more double bonds in the carbon chain structure. Omega-6 fatty acids include linoleic acid (LA), γ-linolenic acid (GLA) and arachidonic acid (AA). Omega-3 fatty acids include eicosapentaenoic acid (EPA) and docosahexaenoic acid (DHA).

Their intake is important since their limited synthesis in humans (Youdim et al., 2000; Fotuhi et al., 2009; Sokoła-Wysoczańska et al., 2018).

Cell-membrane PUFAs composition could be modified with dietary supplementation but it depends on age and probably also on the quantity PUFAs integration (Calder, 2006). High fatty acid diet increases their percentage in inflammatory cell membranes of inflammatory cell and reduces AA levels, a stress-related biomarker and an inflammatory process trigger (through pro-inflammatory eicosanoids production) (Calder, 2002; Dyall, 2015).

Polyunsaturated fatty acids, in particular EPA and DHA, are interesting because of their beneficial effects in preventing cognitive decline through neuroprotective properties such as increasing nerve membrane neuroplasticity, promoting synaptogenesis, modulating signal transduction pathways in neuronal cells, and attenuating inflammatory processes (Youdim et al., 2000; Miller et al., 2017; Sokoła-Wysoczańska et al., 2018).

Furthermore, DHA, produced by the desaturation and elongation of α-linolenic acid (ALA), is able to influence a certain number of membrane proteins, such as receptors, ion channels and enzymes. Furthermore, DHA can modulate dopaminergic, serotonergic, and cholinergic neurotransmission, thus regulating signal transduction pathways (Parletta et al., 2013). DHA is also considered important for neurogenesis regulation, neural synapses increase and neuronal damage protection (Cruz-Jentoft et al., 2019).

In fact, Omega-3 DHA is directly absorbed into cell membranes: it composes at least 30% of the brain matter (in general, fats are more than 50% of the brain) (Parletta et al., 2013). DHA level decreases significantly both in the blood plasma and in the brain, in physiological aging, above all in AD patients (Dupont et al., 2019) because of its lower exogenous intake and its greater oxidation (Yurko-Mauro et al., 2015). However, several studies suggest that Omega-3 fatty acid integration is beneficial only in the early stages of cognitive decline (Parletta et al., 2013).

Indeed, there are discrepancies about fatty acid effectiveness on cognitive functioning (Jiao et al., 2014; Burckhardt et al., 2016; Zhang et al., 2016; Stavrinou et al., 2020). That because of multiple variables such as PUFA amount to administer (both omega-3 and omega-6), the type and quality of their source (such as fish oil and/or vegetable oil or other), differences among tests to investigate cognitive efficiency, sample homogeneity in terms of age and functioning and/or cognitive impairment (Stavrinou et al., 2020). A recent double-blind randomized study investigated the effectiveness of fatty acid intake (omega-3 and omega-6) combined with other antioxidant vitamins in a group of older people with MCI. Neuroaspis PLP10^®^, a nutraceutical containing omega-3 [EPA (810 mg) / DHA (4,140 mg)], omega-6 [GLA (1,800 mg) / LA (3,150 mg)] (1: 1 w / w), vitamin A (0.6 mg) and vitamin E (22 mg as α-tocopherol plus 760 mg as pure γ-tocopherol) was administered to the experimental group subjects for 6 months (Beaudart et al., 2019).

In this study (Beaudart et al., 2019), both tests investigating overall cognitive function (ACE-R and MMSE) showed a significant improvement in the experimental group compared to the control group, regarding memory, language (fluency) and visual-spatial skills (ACE-R). An attentional functionality improvement was evidenced too (specifically, in a symbol cancelation test and in the Stroop test, in particular in the word and color subtests but not in the test in which the interference inhibition capacity is investigated). Besides, from a functional point of view, the experimental group obtained high scores in tests investigating muscle strength, endurance, power, and balance. These physical performance parameters are important since they refer to the most demanding daily activities. In parallel, an increase in the quality of life, sleep and perceived fatigue was demonstrated.

The results of this study are similar to what described by Bo et al. (2017). They showed that 6-month intake of DHA (480 mg/die) and EPA (720 mg/die) could improve the perceptual speed, spatial imagery efficiency, and working memory in MCI elderly. Sinn et al. (2012) has also shown that 6-month intake of fish oils (1.55 g of DHA and 0.40 g of EPA per day) improves cognitive functions and in particular executive efficiency. The same results have not been obtained on patients with known neurodegenerative diseases such as AD, to indicate that greater benefit is drawn from taking PUFA in the early stages of cognitive impairment (Chiu et al., 2008; Cammisuli et al., 2019).

## Coenzyme Q10

Coenzyme Q10 (2,3-dimethoxy-5-methyl-6-decaprenyl-1,4-benzoquinone) is a fat-soluble compound also known as CoQ10, vitamin Q10, ubidecarenone or ubiquinone. An endogenous substance is produced by mitochondria in doses of about 3–5 mg per day. It is one of the main elements involved in mitochondrial oxidative phosphorylation and acts as an antioxidant. *In vitro* studies have shown that CoQ10 easily crosses the blood brain barrier (Somayajulu et al., 2005; Sanoobar et al., 2013).

Thanks to its oxidizing and antioxidant properties, it is a cellular redox state modulator. CoQ10 is located in the internal mitochondrial membrane and protects cells from apoptosis at a morphological and at a molecular level (Beal et al., 1994). Furthermore, as a lipophilic antioxidant, it can eliminate radicals from membranes, cytosol and plasma.

It plays an important role in PD. In fact, CoQ10 levels are significantly lower than normal in neuron and platelet mitochondria of PD patients. *In vitro* studies on fibroblasts of PD patients have shown that CoQ10 intake restores the electron transport chain activity. The first clinical studies on the CoQ10 neuroprotective effects were reported in Beal et al. (1994): this study demonstrated the association between 16-month CoQ10 intake (1,200 mg per day) and a reduced functional decline (44%) in PD patients. Muller et al. (2003) confirmed these data: 28 PD patients showed moderate symptom improvement thanks to CoQ10 oral administration (360 mg per day).

The antioxidant potential of CoQ10 was further evaluated in a pilot study (Chiu et al., 2008) on 11 patients with Rett Syndrome, a severe neurodevelopmental disorder in which hypoxia-induced oxidative stress associates with the pathogenesis and the disease progression (De Felice et al., 2012; Di Pierro et al., 2020). After 12-month CoQ10 intake (300 mg/day), there was a significant improvement in red blood cells’ energy status, suggesting an attenuation of the oxidative stress (De Felice et al., 2012, 2014; Biasini et al., 2018).

Promising results were also observed in a double-blind randomized clinical trial involving patients with remitting-intermittent multiple sclerosis (Sanoobar et al., 2015). The experimental group took 500 mg of CoQ10 for 12 weeks, and showed a significant reduction in inflammatory markers, such as tumor necrosis factor α (TNF-α), interleukin 6 (IL-6) and matrix metalloproteinase 9 (MMP-9).

Ghasemloo et al. (2021) investigate the effect of CoQ10 and miR-149-5p mimic on miR-149-5p, MMPs and Tyrosine hydroxylase in rat PD models. This interaction resulted fundamental to understand how to counteract neurodegeneration in PD: the study showed that the combination of the microRNA miR-149 and CoQ10 was able to prevent the oxidative damage in dopaminergic neurons and improve motor function induced by 6-Hydroxypopamine injection by reducing matrix metalloproteinase 2,9 in an animal model.

## Nigella sativa

*Nigella sativa* L. (*N. sativa*), also known as black cumin, is a plant grown in the Mediterranean countries, in the south and south-west Asia, characterized by its high bioactive-compound content seed (e.g., Tocopherols, vitamin A and C, β-carotene, etc.) and its anti-inflammatory, antioxidant, immunomodulating and anticancer properties (Gholamnezhad et al., 2016; Isik et al., 2017; Ikhsan et al., 2018). *N. sativa* contains fixed oil (22–38%), volatile oil (0.40–1.5%), proteins (21–31%), carbohydrates (25–40%), minerals (3.7–7%), vitamins (1–4%), saponins (0.013%) and alkaloids (0.01%). Its biological activity is associated with its thymoquinone content (TQ) (Bahareh and Hossein, 2016).

Bordoni et al. (2019) revealed the association between the anti-inflammatory and antioxidant properties of *N. Sativa* oil (grown in the Marche region of Italy) and its conservation. Therefore, the Stored Extracted Oil (SEO) and the Fresh Extracted Oil (FEO) were obtained from the same cultivation in order to analyze their thymoquinone content. The cultivated oil showed a higher content of thymoquinone (7,200 mg/mL) compared to other crops (Mohammed et al., 2016; Aziz et al., 2017) and it was higher in FEO while decreased with storage time.

In murine models, it has been demonstrated that thymoquinone is useful to obtain a delayed onset of the microglia degeneration caused by the oxidative stress (Cobourne-Duval et al., 2016). In addition, TQ is able to improve and regenerate antioxidants enzymes such as glutathione peroxidase and glutathione reductase previously repressed by Beta-amyloid in differentiated cell lines of rats affected by AD (Khan et al., 2012).

The mechanisms by which TQ delays neurodegeneration have been clearly elucidated in Parkinson’s disease: it reduces dopaminergic impairment switching on the Nrf2/ARE signaling cascade that triggers the activation of antioxidant genes including Heme Oxygenase 1 (HO-1), Quinone Oxidoreductase (NQO1) and Glutathione-*S*-Transferase (GST) (Dong et al., 2021).

Moreover, an *in vitro* study shows that TQ exerts an inhibition on the α-synuclein aggregation reducing the inflammatory state and improving antioxidant bioavailability (Ardah et al., 2019).

## Chlorogenic Acid

Chlorogenic acid (CA), the main phenolic coffee component, is another polyphenolic substance with an excellent antioxidant activity. It belongs to the chlorogenic acid family (CGA) that are phenolic acids derived from cinnamic acid esterification, such as caffeic, ferulic and p-coumaric acids. The CGA is also widely present in drinks based on herbs, fruits, and vegetables.

Chlorogenic acids have antibacterial, antioxidant and anti-inflammatory activities (Liang and Kitts, 2015). Several *in vitro* and *in vivo* studies have highlighted their ability to counteract neurodegenerative events. Although a preclinical study on AD transgenic mice reported that caffeine reduces brain beta-amyloid (Aβ) levels (Arendash et al., 2006, 2009; Cao et al., 2009), it is still unknown which element is specifically related to AD. Currently, few studies have analyzed CGA effects on human cognitive impairment. Epidemiological studies have found that coffee drinking habits reduce cognitive impairment and the risk of developing neurodegenerative diseases such as AD (Panza et al., 2015; Solfrizzi et al., 2015).

In particular, Kim et al. (2019) investigated the association between coffee intake and AD neuropathological markers *in vivo* (411 healthy elderly subjects).

The results showed that the coffee intake (≥2 cups/day) was associated with lower levels of Aβ brain deposition compared to its less intake (<2 cups/day), suggesting that a moderate daily coffee intake helps to reduce amyloid pathological deposition in the brain (Kim et al., 2019).

Eskelinen et al. (2009) obtained similar results observing that coffee intake in middle age reduces the risk of developing AD in the elderly.

Recently, Kato et al. (Socała et al., 2020) conducted a pilot study and described cognitive function changes after 6-months the CGA intake (330 mg/die) in the elderly with subjective memory loss. Significantly higher scores emerged in tests investigating attentional, executive and mnesic functionality. In the same study, there was a significant reduction in Aβ42, Aβ42 / Aβ40 plasma levels and a significant increase in DHEA-S levels after the CGA intake.

Previous studies have shown that the CGAs improve blood pressure and vascular endothelial functions, both associated with dementia onset (Ota et al., 2010; Kato et al., 2018; Singh et al., 2020): in fact, hypertension, in middle age, is a risk factor for dementia and cognitive impairment in old age and continuous CGA consumption may delay its onset (Ochiai et al., 2004).

Saitou et al. (Watanabe et al., 2006) investigated CGA effects on healthy subjects with subjective memory loss. In this randomized controlled double-blind study, experimental group took a compound based on the CGA caffeoylquinic acids (CQA), feruloylquinic acids (FQA) and dicaffeoylquinic acids (diCQA) for 16 weeks; CQA—FQA total amount was 300 mg, obtained by extraction from green coffee beans. Participants underwent a neuropsychological examination (MMSE and RBANS) at baseline, after 8 weeks and after 16 weeks. At the end of the treatment, significant differences between the CGA intake group and the placebo one was evidenced: in particular, elevated scores were recorded in tests investigating motor speed, psychomotor speed, and executive functions. The serum concentration of cognitive impairment-linked biomarkers revealed an increase in apolipoprotein A1 (ApoA1) and Transthyretin (TTR) levels in the experimental group at 16 weeks (Watanabe et al., 2006).

Considering these results, the CGA intake may improve not only motor activity, but also the cognitive functions that control its execution and monitor its efficiency.

These results confirm what was described previously by the same authors in a pilot study (Eskelinen et al., 2009).

As far as Parkinson’s disease, an *in vitro* model demonstrated that CGA cell pretreatment reduced 6-hydroxydopamine-induced ROS production and cell apoptosis (Elias et al., 2012). In PD murine models, the CGA improves motor skills, mitochondrial activity, and the expression of antiapoptotic genes like Bcl-2 while reduces the activation of the proapoptotic ones (Saitou et al., 2018).

## Selenium

Selenium is an essential micronutrient with a very narrow recommended dietary range. The RDA for selenium is around 55 μg/day and it can be integrated with a specific dietary intake. Selenium, in the form of selenocysteine, is a component of 25 selenoprotein classes, including GPx, selenoproteins P, W and R and thioredoxins (TrxR). As an antioxidant, it provides protection from ROS-induced cellular damage (Brauer and Savaskan, 2004; Xiong et al., 2007; Steinbrenner and Sies, 2013).

Its brain concentration changes in Alzheimer’s disease patients and Multiple Sclerosis ones; therefore, this element may have an important role in the protection from neurodegeneration (Wenstrup et al., 1990; Ceballos-Picot et al., 1996; Clausen et al., 1998; Cornett et al., 1998). Considering that older people are more exposed to selenium deficiency due to metabolic changes, lower bioavailability, and diet changes (Planas et al., 2004; Arnaud et al., 2007; Letsiou et al., 2009), several studies have hypothesized the possibility of its exogenous assumption in order to prevent aging-related diseases.

Selenoproteins, such as glutathione peroxidases (GPx), play an important role in antioxidant defenses. The main brain selenoproteins are P and GPx: the first one has been identified in senile plaques and neurofibrillary tangles, suggesting its important role against oxidative damage (Bellinger et al., 2008; Takemoto et al., 2010), GPx, which neutralizes peroxides, is expressed by neurons and glial cells (Garcia et al., 2009; Zhang et al., 2010). The biosynthesis of selenoproteins depends on selenium availability. Therefore, an adequate selenium intake may be particularly important for maintaining the elderly function (Steinbrenner and Sies, 2013).

Brazil nut (Bertholletia excelsa) is the richest dietary selenium source, and its intake improves selenium status (Thomson et al., 2008; Cominetti et al., 2012). Although some studies have reported that selenium stet is important for maintaining cognitive efficiency (Berr et al., 2000; Gao et al., 2007; Cardoso et al., 2010), only a few studies have evaluated its real clinical efficacy. Cardoso et al. (2010) analyzed the effects of Brazil nut consumption on cognitive function in a group of older people with MCI. The experimental group took a 5-gram Brazil nut per day, containing approximately 288.75 μg of selenium (more than the recommended levels, 55 μg/day, but not exceeding the tolerable upper intake level, 400 μg/day) (Cardoso et al., 2010). Selenium plasma and erythrocyte concentrations, Gpx activity in erythrocytes, ability to absorb oxygen radicals and MDA, and lipid peroxidation genotoxic product were recorded at baseline and after 6 months. The CERAD neuropsychological battery assessed cognitive functions. After 6 months, no selenium deficiency was observed in the treated group, while control subjects had a level below the cut-off (>84–100 μg / L). Furthermore, an increase in plasma and erythrocyte selenium concentrations was observed in the experimental group, there was also a significant improvement in erythrocyte GPX activity. Although no intergroup changes emerged in overall cognitive performance, assessed with the CERAD total score, subtests investigating constructive praxis and verbal fluency showed higher scores in the treated group.

## Probiotics

Probiotics refer to a group of live nonpathogenic microorganisms, which, when administered in adequate amounts, can establish the microbial balance, particularly in the gastrointestinal tract (Wang et al., 2017). Their importance is also related to their antioxidant properties: they act as metal-ion chelators, have their own antioxidant enzymatic systems (SOD and CAT), can produce various metabolites (GSH, butyrate and folate) and mediate Antioxidant Signaling Pathways (Wang et al., 2017).

According to the theory of the “gut-brain axes,” the gut microbiota can have significant effects on cognitive alterations and these alterations can be partially reversed by colonization of the gut (Sudo et al., 2004). Bagga et al. (2018) showed that Probiotic administration for 4 weeks was associated with changes in several brain activation pathways regarding emotional memory and emotional decision-making abilities.

Therefore, a rational manipulation of intestinal microbiota through probiotics, could affect positively Central Nervous System-associated disorders. Bonfili et al. (2018) showed that a probiotic formulation (namely SLAB51) counteracted brain oxidative damages associated with AD. A clinical trial by Kobayashi et al. (2017) investigated the effects of oral administration of *Bifidobacterium breve* strain A1 (*B. breve* A1) on behavior and physiological processes in AD model mice. The consumption of *B. breve* A1 suppressed the hippocampal expressions of inflammation and immune-reactive genes that are induced by amyloid-β suggesting that *B. breve* A1 has therapeutic potential for preventing cognitive impairment in AD.

Michael et al. (2019) investigated the neuroprotective role of two bacterial consortia, known as Lab4 and Lab4b, using the established SH-SY5Y neuronal cell model. Both consortia were equally able to attenuate intracellular reactive oxygen species accumulation in SH-SY5Y cells.

**FIGURE 2:**
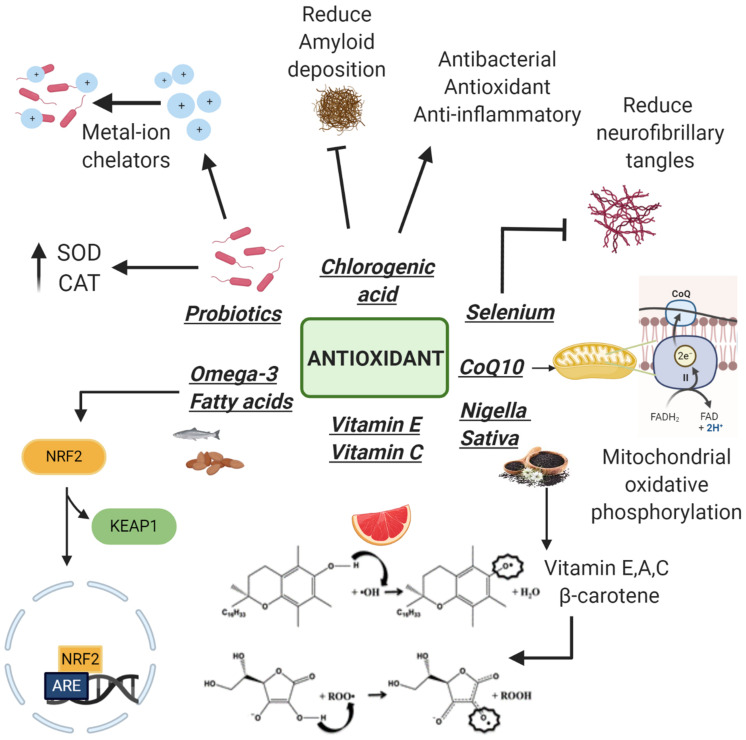
Antioxidants with neuroprotective properties. Following the detachment of Keap1 subunit, Omega-3 increases the antioxidant genes expression. Vitamins E, C, and *Nigella sativa* (rich in vitamins) neutralize free radicals thanks to the presence of an electrophilic hydroxyl group on the chromane ring. Coenzyme Q10 (CoQ10) plays a fundamental role in the electron transport chain protecting cells from apoptosis at a morphological and molecular level. Selenium is able to reduce neurofibrillary tangle formation while chlorogenic acid reduces amyloid deposition. Probiotics act as metal ion chelators and as antioxidants using their antioxidant enzyme systems: superoxide dismutase and catalase (SOD and CAT).

Another clinical trial showed that heat-killed *L. buchneri* KU200793 has an important antioxidant activity mediated by its ability to increase levels of BDNF and so its intake can be considered useful in PD prevention (Cheon et al., 2020). Therefore, in accordance with the above, thanks to their antioxidant properties, probiotics seems to be fundamental to delay the progression of these neurodegenerative disorders (Figure 2).

## Conclusion

Lots of natural compounds contain antioxidant molecules that are protective against free radical damage affecting brain cells. *In vitro* and murine models have widely demonstrated that antioxidant improve oxidative stress status of brain cells, cognitive functions and motor skills. Further clinical trials should be conducted in order to understand if these natural compounds, alone or in combination with an appropriate pharmacological treatment, can effectively delay the potential onset of neurodegenerative disorders and ameliorate brain functions. Moreover, it should be better elucidated the actual bioavailability in the central nervous system of these natural antioxidants, and their effective ability to pass the blood brain barrier after an oral intake.

## Author Contributions

MA and JF: formal analysis of scientific literature. FF, GS, and SG: writing—original draft preparation. FF and GS: writing—review and editing. CP: supervision. All authors have read and agreed to the published version of the manuscript.

## Conflict of Interest

The authors declare that the research was conducted in the absence of any commercial or financial relationships that could be construed as a potential conflict of interest.

## Publisher’s Note

All claims expressed in this article are solely those of the authors and do not necessarily represent those of their affiliated organizations, or those of the publisher, the editors and the reviewers. Any product that may be evaluated in this article, or claim that may be made by its manufacturer, is not guaranteed or endorsed by the publisher.
